# Incidence of acute hemorrhagic conjunctivitis in Chongqing: a forecasting study based on mathematical models

**DOI:** 10.3389/fpubh.2025.1644729

**Published:** 2025-10-10

**Authors:** Xiaobing Xian, Sitian Wu, Yandi Fu, Xiaoli Fan, Yan Cheng, Li Zeng, Zhangmei Hou, Yinzhi Chen

**Affiliations:** ^1^Department of Operations Management, The Thirteenth People's Hospital of Chongqing, Chongqing, China; ^2^Department of Operations Management, Chongqing Geriatrics Hospital, Chongqing, China; ^3^School of Pediatric, Chongqing Medical University, Chongqing, China; ^4^College of public health, Chongqing Medical University, Chongqing, China; ^5^Department of Healthcare-associated Infection Control, Chongqing General hospital, Chongqing University, Chongqing, China; ^6^School of Mathematics and Statistics, Chongqing Technology and Business University, Chongqing, China

**Keywords:** acute hemorrhagic conjunctivitis, SARIMA, prophet, SARIMA-KNN, SARIMA-Prophet

## Abstract

**Background:**

Acute hemorrhagic conjunctivitis (AHC) is a highly infectious eye disease. It poses a significant threat to public health given its propensity for rapid transmission in densely populated areas. Recent epidemiological data have demonstrated a distinct seasonal outbreak pattern in Chongqing. However, conventional single prediction models exhibit limitations in accurately capturing the complex spatiotemporal transmission characteristics of AHC. This study endeavors to compare the performance of different mathematical models in forecasting AHC incidence in Chongqing. Through the investigation of optimal predictive methodologies, this study establishes a theoretical foundation for relevant department to formulate policies for preventing AHC.

**Methods:**

The monthly incidence data of AHC in Chongqing from March 2019 to October 2024 were collected from the official website of the Chongqing Municipal Health Commission. Five predictive models (SARIMA, KNN, Prophet model as well as SARIMA-KNN and SARIMA-Prophet model) were employed to fit the incidence data. The data from March 2019 to December 2023 was designated as the training set, while the data from January 2024 to October 2024 served as the test set. Model performance was evaluated through multiple metrics, including MSE, RMSE, MAE, and MAPE. Subsequently, the Diebold-Mariano test was implemented to statistically assess the significance of predictive performance differences among the five models.

**Results:**

During the period from March 2023 to October 2024, the incidence rate of AHC in Chongqing showed a pronounced seasonal fluctuation pattern, with the peak period consistently occurring between June and September annually. The comparative analysis of model performance revealed that the SARIMA-KNN hybrid model demonstrated optimal performance metrics in terms of MSE, MAE, RMSE, and MAPE. Furthermore, the predicted curve of the SARIMA-KNN model demonstrated superior fitting accuracy compared to the actual curve. The Diebold-Mariano statistical test confirmed that the SARIMA-KNN model's performance was significantly superior to other models.

**Conclusion:**

In comparison with the other four models, the SARIMA-KNN hybrid model effectively integrates the temporal characteristics of AHC incidence. It offers the technical support for the development of early warning systems and the formulation of prevention and control strategies in Chongqing. This approach holds substantial practical significance in the field of public health.

## 1 Introduction

Acute hemorrhagic conjunctivitis (AHC) is a highly contagious viral conjunctivitis primarily caused by enterovirus 70 (EV70) or coxsackievirus A24 (CVA24) infection (1). Its typical clinical symptoms include conjunctival hyperemia, photophobia, epiphora, and foreign body sensation (2). Owing to its short incubation period and high infectivity, AHC is prone to rapid outbreaks and epidemics (3). Since its initial identification in Ghana in 1969 (4), AHC has shown periodic epidemic patterns globally, with significant prevalence observed in Asia, Africa, and Latin America (5). In China, the first outbreak of AHC was reported in Hong Kong in 1971 (6). As one of the most prevalent ocular infectious diseases in China, AHC has been reported in numerous cities. From 2005 to 2012, Chongqing consistently ranked among the top five regions nationwide in terms of AHC incidence rates (5). Recent monitoring shows that Chongqing persists as one of China's high-incidence regions for AHC. According to monitoring reports from the China CDC Information System (CIDCIS), the national incidence rate of AHC showed periodic fluctuations from 2013 to 2020, with 2014 and 2019 being the peak years of reporting. A notable trend is that after 2020, due to the high-intensity COVID-19 prevention and control measures, the reported incidence rate decreased significantly (7). However, with the full resumption of societal activities, epidemic intensity has manifested a rising trend. This phenomenon highlights the urgency of strengthening local prevention and control and prediction research on AHC after the epidemic.

The transmission of AHC depends on multiple factors, including climatic conditions (temperature, humidity) (8), population mobility patterns, and sanitation infrastructure (9, 10). Within Chongqing's subtropical monsoon climate, the virus transmission is potentially exacerbated by the hot and rainy summer conditions coupled with cold and humid winter environments (11). In addition, as a large-scale city in the central-southern region, the substantial population mobility significantly increases the risk of disease transmission. Recent epidemiological studies have demonstrated that the incidence of AHC in Chongqing has shown a cyclical fluctuation and sudden growth trend (12). The current infectious disease early warning and monitoring platforms mainly rely on traditional statistical models. These models demonstrate limited sensitivity in forecasting diseases with sudden and non-linear transmission characteristics. Meanwhile, existing platforms struggle to dynamically integrate heterogeneous data such as climate and population mobility (13). The accurate detection and early warning of AHC mixed infections remain challenges.

In recent years, advances in infectious disease prediction models have shifted from single statistical methodologies to multi-model integration and data-driven approaches. This transition aims to address the complex characteristics of medical data by leveraging the strengths of diverse algorithms (14). Notably, hybrid models that combine classical statistical methods with machine learning techniques have exhibited remarkable advantages in forecasting various infectious diseases, including tuberculosis (15), hepatitis B (16), and hand-foot-mouth disease (17). Yet research on AHC remains scarce, especially in the context of Chongqing, a city with a complex climate and a dense population, where systematic exploration has yet to be conducted.

The Seasonal Autoregressive Integrated Moving Average Model (SARIMA), an extension of the ARIMA framework, has gained widespread recognition in time series forecasting applications (18). The fundamental principle of SARIMA involves eliminating non-stationarity within the sequence through difference operations while incorporating seasonal parameters (P, D, Q, S) to effectively capture periodic patterns. This model demonstrates superior performance in forecasting periodic data. The K-Nearest Neighbors (KNN) algorithm is generally used for basic classification and regression analysis. In regression methods, this algorithm relies on the k nearest dependent variable values to predict a given data (19). The distance between two data points can be determined using a distance function (20). It is often used to capture local non-linear fluctuations and short-term trends. In most cases, as linear models cannot produce sufficient results, non-linear structures are adopted in time series analysis (21). We also introduce Prophet, a time series forecasting method based on an additive model developed by Facebook. Its core is to perform curve fitting within the Bayesian inference framework to achieve smoothing and prediction of time series data (22). This model shows robust performance in handling missing values and accommodating trend changes, while effectively fit complex multiple seasonal patterns.

SARIMA model excels in handling seasonality and trends within time series (23). KNN model demonstrates flexibility in generating accurate predictions based on local data characteristics (24). Concurrently, the Prophet model demonstrates superior performance in managing complex seasonality, trend variations, and outlier detection. To enhance the handling of intricate time series data and improve predictive accuracy, this study proposes the SARIMA-KNN hybrid model for the first time, and also introduces the SARIMA-Prophet hybrid model based on the SARIMA and Prophet models. In the predictive process, SARIMA is initially employed to extract linear components from the time series, followed by the application of KNN and Prophet models to model the residual sequences from SARIMA, thereby capturing non-linear features in the data. This multi-model integration strategy enables the simultaneous utilization of diverse algorithmic advantages, offering a more comprehensive representation of complex time series characteristics.

In conclusion, this study conducted a systematic comparison of the predictive performance among three single models and two hybrid models for forecasting the incidence of AHC in Chongqing. The research aims to identify the most effective predictive methodology and establish a theoretical foundation for early warning systems and resource allocation strategies pertaining to AHC in Chongqing.

## 2 Material and methods

### 2.1 Data

This study utilized the AHC data published by the Chongqing Municipal Health Commission (https://wsjkw.cq.gov.cn/) from March 2019 to October 2024. The Chongqing Municipal Health Commission is the official municipal health authority, and the data has been strictly reviewed, ensuring its authority and reliability. In terms of data quality control, all case diagnoses were made in accordance with the national unified “Diagnosis Criteria for Acute Hemorrhagic Conjunctivitis”, ensuring consistency in diagnostic standards. Additionally, the Chinese government attaches great importance to the monitoring of legally notifiable infectious diseases and implements a system of “local management and hierarchical responsibility”. AHC data is reported by grassroots medical institutions within 24 h of diagnosis and is successively reviewed and monitored by disease prevention and control institutions at the district (county), municipal, provincial, and national levels before being released by the Health Commission. This ensures the timeliness and accuracy of the data. The data does not involve personal information, so no professional ethical review is required.

**Figure 1:**
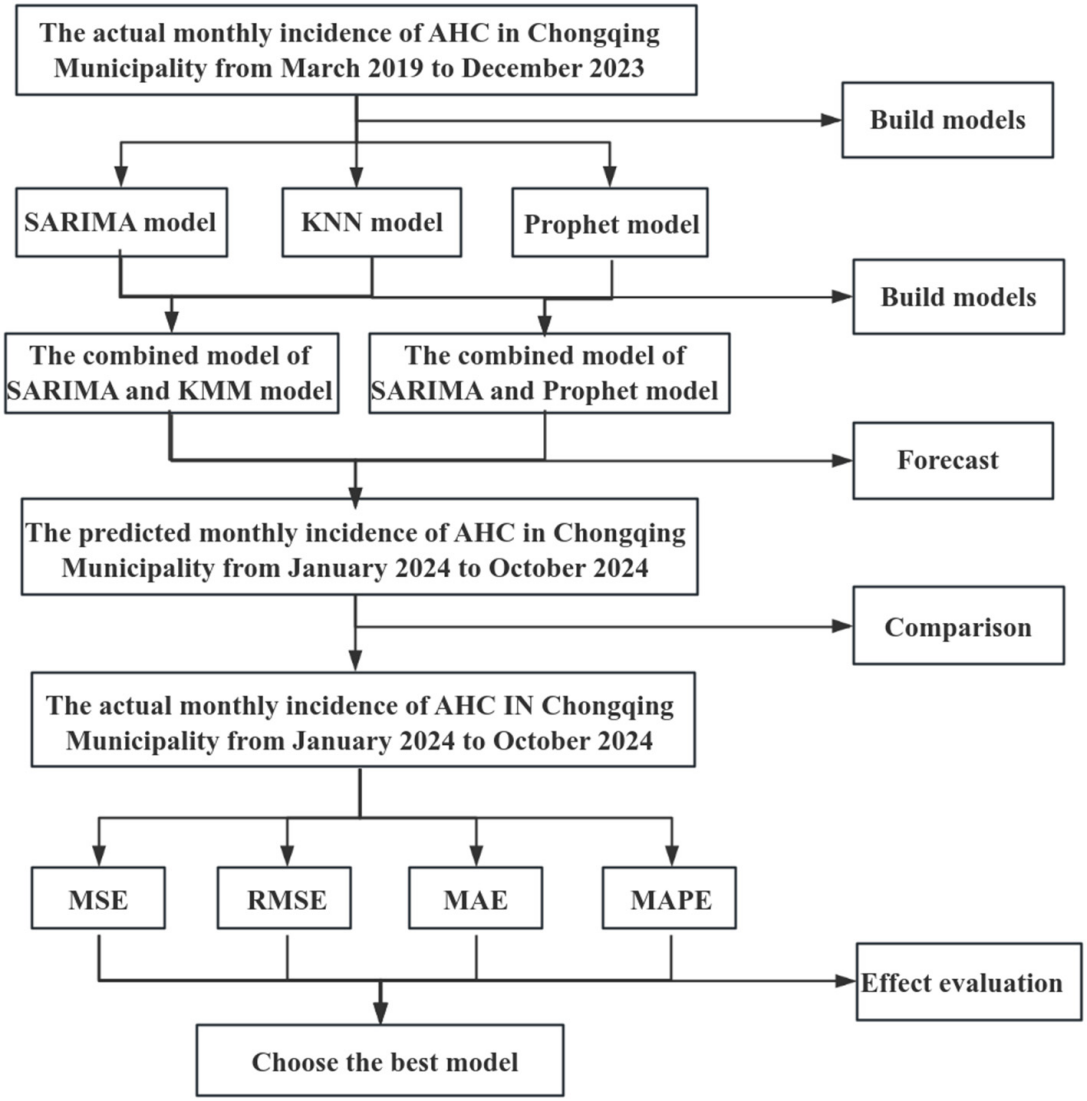
Technical route diagram of the development, prediction, and evaluation process for the AHC incidence prediction model in Chongqing.

This study employed the SARIMA model, KNN model, Prophet model, and two combined models to fit the monthly incidence rate of AHC. The incidence rate from March 2019 to December 2023 was used as the training set. The incidence rate from January 2024 to October 2024 was used as the test set to validate the predictive performance of the five models. The technical route diagram is shown in Figure 1.

### 2.2 Data analysis software

Data preprocessing and descriptive statistics were conducted using SPSS 25.0. Model fitting procedures for SARIMA, KNN, Prophet models, along with SARIMA-KNN and SARIMA-Prophet models were implemented in R 4.3.0. Throughout the study, statistical significance was determined at the conventional threshold of *P* < 0.05.

### 2.3 SARIMA model

The SARIMA model, as an extension of the ARIMA model, is specifically designed to handle time series data with seasonal components (25). The structure of a complete SARIMA model is expressed as:


(1) $$\begin{array}{l} \text{SARIMA}\left(\text{p},\text{d},\text{q}\right)\times \left(\text{P},\text{D},\text{Q}\right)\text{m} \end{array}$$

where p, d, and q represent the autoregressive order, the differencing order, and the moving average order of the non-seasonal part, respectively; P, D, and Q are the corresponding orders of the seasonal part; and m indicates the length of the seasonal cycle (for example, *m* = 12 for monthly data) (26). The general mathematical representation of the SARIMA model is as follows:


(2) $$\begin{array}{l} \Phi_{\text{p}}\left({\text{B}}^{\text{m}}\right)\varphi_{\text{p}}\left(\text{B}\right){\left(1-{\text{B}}^{\text{m}}\right)}^{\text{D}}{\left(1-\text{B}\right)}^{\text{d}}{\text{y}}_{\text{t}}=\theta_{\text{Q}}\left({\text{B}}^{\text{m}}\right)\theta_{\text{q}}\left(\text{B}\right)\omega_{\text{t}} \end{array}$$

B is the backward shift operator, *y*_*t*_ is a non-stationary time series, ω_*t*_ is a Gaussian white noise process. D is the seasonal difference term, and D = 1 is sufficient to enforce data stationarity. φ_*p*_(*B*) is the non-seasonal autoregressive polynomial, θ_*q*_(B) is the non-seasonal moving average polynomial, $\Phi_{p}\left(B^{m}\right)$ is the seasonal autoregressive polynomial, and $\Theta_{Q}\left(B^{m}\right)$ is the seasonal moving average polynomial. The expression of the four-term polynomial is as follows:


(3) $$\begin{array}{l} \varphi_{\text{p}}\left(\text{B}\right)=1-\varphi_{1}\text{B}-\varphi_{2}{\text{B}}^{2}-\cdots -\varphi_{\text{p}}{\text{B}}^{\text{p}} \end{array}$$

(4) $$\begin{array}{l} \theta_{\text{q}}\left(\text{B}\right)=1+\theta_{1}\text{B}+\theta_{2}{\text{B}}^{2}+\cdots +\theta_{\text{q}}{\text{B}}^{\text{q}} \end{array}$$

(5) $$\begin{array}{l} \Phi_{\text{P}}\left({\text{B}}^{\text{m}}\right)=1-\Phi_{1}{\text{B}}^{\text{m}}-\Phi_{2}{\text{B}}^{2\text{m}}-\cdots \;\cdots -\Phi_{\text{P}}{\text{B}}^{\text{Pm}} \end{array}$$

(6) $$\begin{array}{l} \theta_{\text{Q}}\left({\text{B}}^{\text{m}}\right)=1+\Theta_{1}{\text{B}}^{\text{m}}+\Theta_{2}{\text{B}}^{2\text{m}}+\cdots \;\cdots +\theta_{\text{Q}}{\text{B}}^{{\text{Q}}_{\text{m}}} \end{array}$$

The construction of a SARIMA model mainly involves three steps: stationarity test, model selection, and parameter verification.

Firstly, conduct a stationarity test on the original sequence. Use the Augmented Dickey-Fuller (ADF) unit root test; if *p* < 0.05, the sequence is considered stationary. If not, perform difference: typically, start with seasonal differencing, and if it remains non-stationary, proceed with non-seasonal difference. Additionally, identify the presence of seasonality and the value of the cycle m by plotting the sequence graph, seasonal decomposition graph, and calculating the periodic autocorrelation.

Secondly, for the stationary sequence, plot its autocorrelation function (ACF) and partial autocorrelation function (PACF) graphs. The ACF quantifies the correlation between a time series and its lagged values, while the PACF measures the correlation between the time series and its lagged values at a specific time interval, excluding the influence of intermediate lags (27). The ACF of a time series can be expressed as:


(7) $$\begin{array}{l} \text{ACF}\left({\text{y}}_{\text{t}},{\text{y}}_{\text{t}-\text{k}}\right)=\frac{\text{Covariance}\left({\text{y}}_{\text{t}},{\text{y}}_{\text{t}-\text{k}}\right)}{\text{Variance}\left({\text{y}}_{\text{t}}\right)} \end{array}$$

K is the lag period, defined as the difference between *y*_*t*_ and *y*_*t*−*k*_.

The PACF between two observations can be expressed as follows:


(8) $$\begin{array}{l} \text{PACF}\left({\text{y}}_{\text{t}},{\text{y}}_{\text{t}-2}\right)=\frac{\text{Covariance}\left({\text{y}}_{\text{t}},{\text{y}}_{\text{t}-2}|{\text{y}}_{\text{t}-1}\right)}{\sqrt{\text{Variance}\left({\text{y}}_{\text{t}}|{\text{y}}_{\text{t}-1}\right)}\sqrt{\text{Variance}\left({\text{y}}_{\text{t}-2}|{\text{y}}_{\text{t}-1}\right)}} \end{array}$$

Based on the truncation and drag tail characteristics observed in the ACF/PACF plots, the preliminary estimation of the parameters p, q, P, and Q can be established as follows:

The order of the non-seasonal AR term p: PACF truncates after lag p.
The order of the non-seasonal MA term q: ACF truncates after lag q.
The order of the seasonal AR term P: PACF truncates at seasonal lags (such as *m*, 2*m*, ...).
The order of the seasonal MA term *Q*: ACF truncates at seasonal lags.

After the initial determination of the order, the model parameters are fitted using the maximum likelihood estimation (MLE) or conditional least squares estimation method. Multiple candidate models are compared using information criteria such as AIC, AICc, and BIC, and the model with the smallest value is preferred.


(9) $$\begin{array}{l} \text{AIC}=-2\log\text{L}\left(\hat{\theta }\right)+2\text{K} \end{array}$$

(10) $$\begin{array}{l} \text{AICc}=-2\text{ log L}\left(\hat{\theta }\right)+\text{2K}+\frac{\text{2K}\left(\text{K}+\text{1}\right)}{\text{N}-\text{K}-\text{1}} \end{array}$$

(11) $$\begin{array}{l} \text{BIC}=-2\text{ log L}\left(\hat{\theta }\right)+\text{K log N} \end{array}$$

$\mathrm{Log}L\left(\hat{\theta }\right)\;$represents the likelihood function, *K* indicates the total number of model parameters, and *N* is the quantity of observed data.

Third, parameter testing: The Ljung–Box *Q* test is used to test the white noise residuals. If *P* > 0.05, it indicates a white noise sequence, confirming that the model effectively captures the data information and its validity is statistically significant. Conduct *t*-statistic tests on the model parameters. *P* < 0.05 indicates statistical significance, suggesting that we consider the established model to be appropriate.

### 2.4 KNN model

The principle of KNN for time series prediction is based on “K nearest similarity.” The core methodology involves identifying the most similar historical segments to the current time window and use the subsequent values of these similar segments for prediction (28). The steps are as follows:

First, the original data is transformed into a structured representation suitable for supervised learning through preprocessing. In this study, the sliding window method is used to reconstruct the continuous time series, dividing the original sequence *X* = (*x*_1_, *x*_2_,..., *x*_*T*_) of length *T* into several fixed-length observation windows with *n* = 12. For each time point *t*, its feature vector is defined as *S*_*t*_ = (*x*_*t*−*n*+1_, *x*_*t*−*n*+2_,...,*x*_*t*_), with the corresponding target output being the future h = 1 step sequence segment Y_t = (x_(t+1), x_(t+2), ..., x_(t + h)). To further enhance the feature representation ability, sliding statistics can be introduced as auxiliary features to construct a multi-dimensional feature space.

Next, calculate the distance between the target sample and each individual instance within the training data. Common distance measurement methods include Euclidean distance, Manhattan distance, Minkowski distance (29, 30).

The Minkowski distance used in this study is a generalization of the Euclidean distance and the Manhattan distance, with the formula:

*d* = ${\left(\overset{n}{\underset{i=1}{\sum }}|x_{1i}-x_{2i}{|}^{p}\right)}^{\frac{1}{p}}$ (When *p* = 2, it is the Euclidean distance; when *p* = 1, it is the Manhattan distance.)

Then, based on the calculated distances, select the *K* sample points in the training set that are closest to the test sample, and vote or average the values of these points to obtain the prediction result.

Finally, make adjustments and optimizations, using 5-fold cross-validation and grid search for model training and parameter tuning.

### 2.5 Prophet model

The Prophet model, developed by Facebook, is an advanced time series data, this model employs a Bayesian-based curve fitting methodology to both smooth and predict time series data, thereby facilitating the rapid acquisition of desired forecasting outcomes. The Prophet model comprises three principal components: trend, seasonality, and holidays (22). Its fundamental equation is formulated as follows:


(12) $$\begin{array}{l} \text{y}\left(\text{t}\right)=\text{g}\left(\text{t}\right)+\text{s}\left(\text{t}\right)+\text{h}\left(\text{t}\right)+{\text{ε}}_{\text{t}} \end{array}$$

Here, g(t) denotes the trend function characterizing the non-periodic variations in the time series, s(t) represents the seasonal component, h(t) signifies the influence of holidays or specific events on the time series, and ϵ(t) constitutes the error term (31).

Regarding the trend modeling approach, it encompasses the fitting of piecewise linear curves or non-linear saturation growth models. The growth pattern is conventionally modeled through the logistic growth model, whose fundamental formulation is as follows:


(13) $$\begin{array}{l} \text{g}(\text{t})=\frac{\text{C}}{1+\text{exp}(-\text{k}(\text{t}-\text{m}))} \end{array}$$

In this context, C denotes the carrying capacity, k signifies the growth rate, and m indicates the offset parameter. Notably, both the carrying capacity and the growth rate are non-constant variables. Through parameter rate adjustment, the model's flexibility can be effectively modulated.

### 2.6 SARIMA-KNN model

The SARIMA-KNN model integrates the advantages of the SARIMA algorithm and the KNN algorithm through a two-stage modeling strategy. Firstly, a SARIMA (p, d, q)(P, D, Q)s model is constructed. The non-stationarity of the sequence is eliminated through difference operations (d, D), and the deterministic structural features of the time series are extracted using autoregressive (p, P), moving average (q, Q), and seasonal (s) components. Then, the standardized residual sequence derived from the SARIMA model is utilized as input for the KNN model. This residual term contains the non-linear features and random components in the original sequence that were not explained by the linear model. By optimizing the length L of the local modeling window through an adaptive sliding window mechanism, combining a density-sensitive K value selection strategy, and using k-fold cross-validation, the optimal hyperparameter combination is determined through network optimization methods to effectively capture the non-linear dynamic features in the residual sequence.

### 2.7 SARIMA-Prophet model

The SARIMA-Prophet model shares a similar modeling framework with the SARIMA-KNN hybrid model described in Section 2.6, as both employ a phased modeling approach. In the initial phase, both models utilize the SARIMA model for time series fitting and forecasting, obtaining the initial prediction results and the residual sequence. The difference is that this model inputs the standardized residual sequence into the Prophet model. The Prophet model automatically decomposes and fits the complex seasonal patterns and non-linear trend changes in the residuals through an additive model structure. The final prediction is obtained by integrating the forecast outputs from both the SARIMA model and the Prophet model's residual predictions.

### 2.8 Model evaluation

We used root mean square error (RMSE), mean absolute error (MAE), mean absolute percentage error (MAPE), and mean square error (MSE) to evaluate the prediction efficiency of the SARIMA, KNN, and SARIMA-KNN models (32). These indicators measure prediction accuracy from different perspectives:

1) RMSE: sensitive to outliers, with units consistent with the original data, suitable for scenarios where high bias needs to be controlled.
2) MAE: reflects the mean of absolute errors, with strong robustness and not affected by extreme values.
3) MAPE: measures relative error in percentage form, but requires non-zero actual values.
4) MSE: A commonly used objective function for model optimization, but should be combined with RMSE for auxiliary interpretation.

According to previous studies, when the RMSE, MAE, MAPE, and MSE of a model are smaller, the model's goodness of fit is better. The following are the calculation methods:


(14) $$\begin{array}{l} \text{RMSE}=\sqrt{\frac{\sum_{i=1}^{n}{\left(y_{i}-{\hat{y}}_{i}\right)}^{2}}{n}} \end{array}$$

(15) $$\begin{array}{l} \text{MAE}=\frac{1}{\text{n}}\overset{\text{n}}{\underset{\text{i}=1}{\sum }}\left|y_{i}-{\hat{y}}_{i}\right| \end{array}$$

(16) $$\begin{array}{l} \text{MAPE}=\frac{1}{\text{n}}\overset{\text{n}}{\underset{\text{i}=1}{\sum }}|\frac{\left(y_{i}-{\hat{y}}_{i}\right)}{y_{i}}|\times 100\% \end{array}$$

(17) $$\begin{array}{l} \text{MSE}=\frac{1}{\text{n}}{\sum_{\text{i}=1}^{\text{n}}\left(y_{i}-{\hat{y}}_{i}\right)}^{2} \end{array}$$

Furthermore, to further evaluate whether the differences in the predictive performance of the five models are statistically significant, we conducted the Diebold-Mariano test, comparing SARIMA-KNN with each of the other four models one by one, to clarify the statistical differences in the predictive accuracy of different models.

## 3 Results

### 3.1 Descriptive statistical results

**Figure 2:**
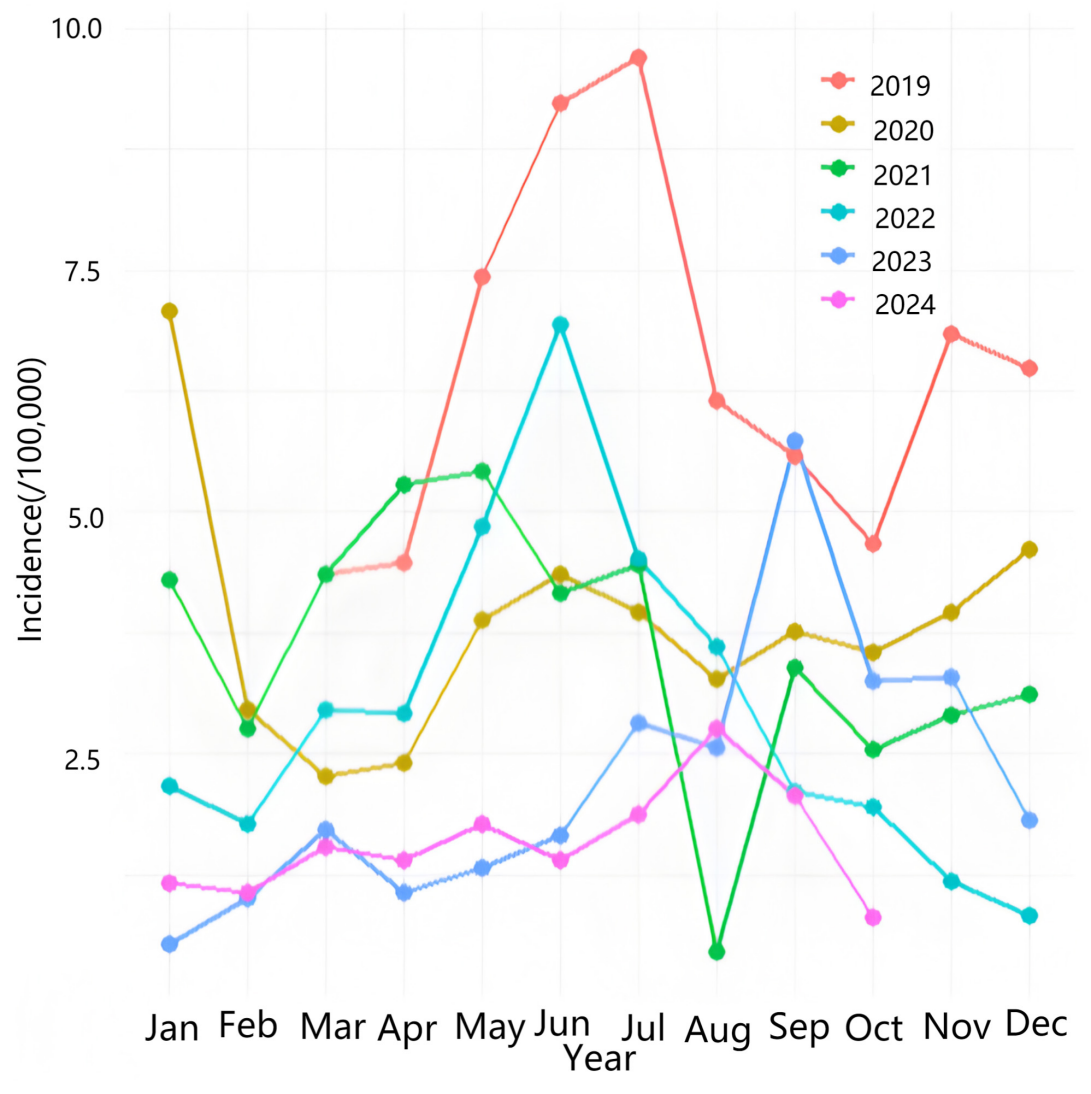
Seasonal distribution of AHC in Chongqing Municipality. The vertical axis represents incidence rate (per 100,000 population), and the horizontal axis indicates months. Lines of different colors correspond to each year, showing distinct seasonal fluctuations with an annual peak between June and September. The magnitude of variation differs across years.

Table 1 shows the monthly incidence rate of acute hemorrhagic conjunctivitis in Chongqing from March 2019 to October 2024. The highest incidence month was July 2019, with a rate of 9.69, while the lowest incidence month was January 2023, with a rate of 0.53. Figure 2 indicates that the incidence rate of AHC in Chongqing is relatively high from June to September each year.

**Table 1:** The incidence distribution of acute hemorrhagic conjunctivitis in Chongqing from March 2019 to October 2024.

**Year**	**Month (Incidence/Hundred Thousand)**											
	**1**	**2**	**3**	**4**	**5**	**6**	**7**	**8**	**9**	**10**	**11**	**12**
2019			139 (4.36)	143 (4.49)	237 (7.43)	294 (9.22)	309 (9.69)	196 (6.15)	178 (5.58)	149 (4.67)	218 (6.84)	207 (6.49)
2020	227 (7.07)	95 (2.96)	73 (2.27)	77 (2.40)	125 (3.90)	140 (4.36)	127 (3.96)	105 (3.27)	121 (3.77)	114 (3.55)	127 (3.96)	148 (4.61)
2021	138 (4.30)	89 (2.77)	140 (4.36)	170 (5.29)	174 (5.42)	134 (4.17)	143 (4.45)	15 (0.47)	109 (3.39)	82 (2.55)	93 (2.90)	100 (3.11)
2022	70 (2.18)	57 (1.77)	95 (2.96)	94 (2.93)	156 (4.85)	223 (6.94)	145 (4.51)	116 (3.61)	68 (2.12)	63 (1.96)	38 (1.18)	27 (0.84)
2023	17 (0.53)	32 (1.00)	55 (1.72)	34 (1.07)	42 (1.32)	53 (1.66)	90 (2.82)	82 (2.57)	183 (5.73)	104 (3,26)	105 (3.29)	58 (1.82)
2024	37 (1.16)	34 (1.07)	49 (1.54)	45 (1.41)	57 (1.79)	45 (1.41)	60 (1.88)	88 (2.76)	66 (2.07)	26 (0.81)		

### 3.2 Performance of SARIMA model

**Figure 3:**
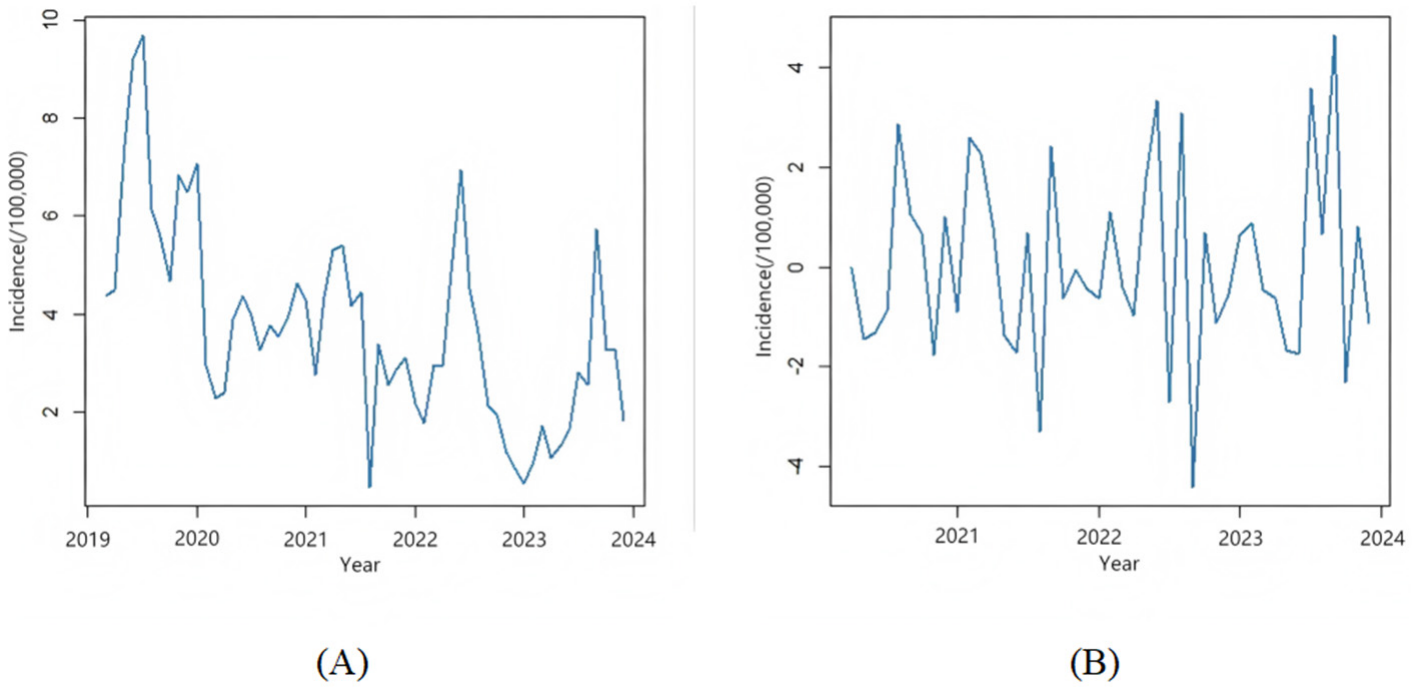
Time series analysis of acute hemorrhagic conjunctivitis incidence. **(A)** Time series plot of the incidence rate of acute hemorrhagic conjunctivitis. **(B)** Time series plot of the sequence after first-order difference and seasonal difference.

**Figure 4:**
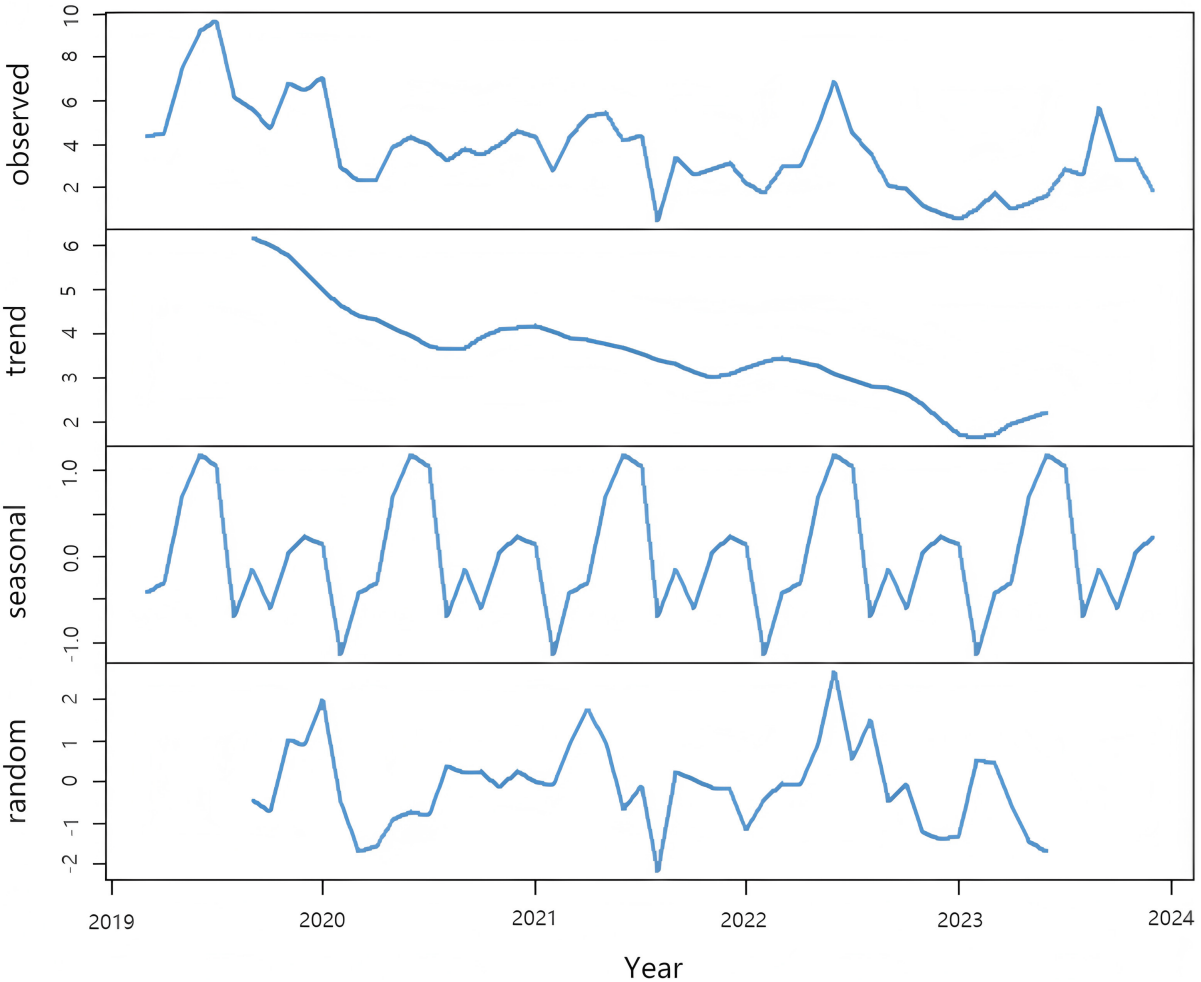
Decomposition of the incidence sequence of acute hemorrhagic conjunctivitis, revealing the underlying trend, seasonality, and random variations in incidence dynamics.

From the time series plot in Figure 3A, it can be observed that the sequence has a clear downward trend, suggesting that it is a non-stationary time series. The ADF test yields *P* = 0.1485 > 0.05. As shown in the seasonal component in Figure 4, the sequence has a distinct seasonal feature with each year as a cycle. Since the data is monthly, the cycle length is 12. After performing first-order differencing and first-order seasonal differencing on the original sequence, the random fluctuations of the sequence become relatively stable, as depicted in Figure 3B. The ADF test results in *P* = 0.01 < 0.05, thus SARIMA (p, 1, q) × (P, 1, Q)12 is initially selected. Through stepwise parameter estimation of the SARIMA model, SARIMA(0, 1, 3) × (P, 1, Q,)12 is ultimately determined as the best prediction model. The Ljung-Box test yields *P* = 0.1292 > 0.05, accepting the null hypothesis, indicating that the fitted model is significantly effective.

### 3.3 Performance of KNN Model

The performance of the KNN model was evaluated using 5-fold cross-validation and grid optimization for model training and parameter tuning. RMSE was used as the criterion for selecting the best model. Through systematic analysis, it was determined that the minimal RMSE value of ~1.610 was achieved when the parameter k was set to 8. Consequently, this specific parameter configuration (k = 8) was identified as the optimal setting for the KNN model, demonstrating its capability to deliver superior predictive accuracy and robust fitting performance for the AHC dataset.

### 3.4 Performance of Prophet model

**Figure 5:**
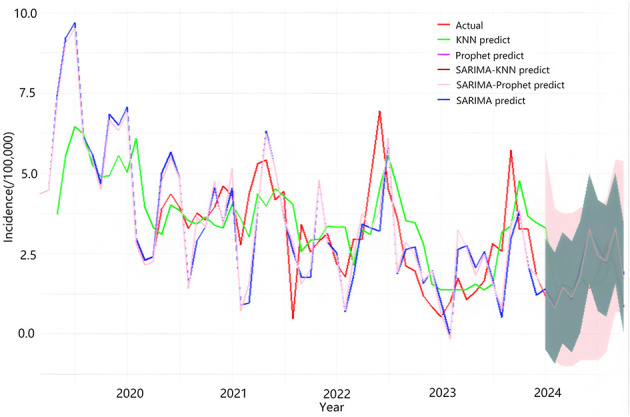
The fitting situation of the actual incidence rate of acute hemorrhagic conjunctivitis from March 2019 to December 2023 and the predicted incidence rate from January 2024 to October 2024. The SARIMA-KNN predicted curve closely aligns with the actual incidence, demonstrating the model's accuracy.

The Prophet model was employed to automatically fit the incidence rate of AHC in Chongqing from March 2019 to December 2023, subsequently projecting the incidence rate from January to October 2024. The predictive outcomes are presented in Table 3, while the fitting and prediction curves are illustrated in Figure 5. The findings demonstrate that the monthly incidence rate of AHC in Chongqing exhibits a distinct seasonal pattern.

### 3.5 Performance of the SARIMA-KNN Hybrid Model

We extracted the deterministic structural features of the sequence using the SARIMA model, and then input the residual sequence of the SARIMA model into the KNN model. We determined the optimal parameters of the hybrid model using the same steps as when modeling separately. Table 2 presents the performance evaluation metrics of the SARIMA-KNN model.

**Table 2:** Evaluation indicators of the three models.

**Evaluation indicators**	**SARIMA**	**KNN**	**SARIMA-KNN**	**Prophet**	**SARIMA-Prophet**
MSE	0.71	1.17	0.62	1.27	1.93
MAE	0.66	0.77	0.63	0.93	1.16
RMSE	0.84	1.08	0.79	1.13	1.39
MAPE	46.28	63.56	43.77	59.88	85.56

MSE, means square error; MAE, mean absolute error; RMSE, root means square error; MAPE, mean absolute percentage error; SARIMA, seasonal autoregressive integrated moving average; KNN, K-nearest neighbors; Prophet, Facebook Prophet, an additive regression model developed by Meta for time series forecasting.

### 3.6 Performance of the SARIMA-Prophet Hybrid Model

The hybrid model first captures the linear trend and seasonal components in the time series through SARIMA, and then inputs the obtained residual sequence into the Prophet model for further fitting of the implicit non-linear features. The performance indicators of the model are shown in Table 2.

### 3.7 Performance Comparison

The performance metrics of all models are presented in Table 2. It is not difficult to see that the SARIMA-KNN model demonstrates lower values in MSE, MAE, RMSE, and MAPE compared to the other four models. The residual error accumulation observed in the SARIMA-Prophet combination further substantiates the rationale for employing KNN to correct non-linear residuals. Furthermore, statistical analysis of predictive performance differences through the Diebold-Mariano test reveals that the SARIMA-KNN combined model exhibits significantly superior prediction accuracy at a statistical level (*P* < 0.05) compared to the remaining models. Consequently, we conclude that the SARIMA-KNN model demonstrates superior accuracy and applicability in predicting the incidence of AHC in Chongqing Municipality.

The prediction results of the SARIMA (0,1,3) × (1, 1, 0)12 model, the KNN model, and the SARIMA-KNN model for January 2024 to October 2024 are shown in Table 3. (The negative value range indicates that individual point predictions should be interpreted with caution, but the overall performance of the model still meets the requirements for early warning) Empirical analysis reveals that the SARIMA-KNN model demonstrates superior predictive accuracy, exhibiting lower prediction errors compared to the other two models across the majority of the predicted months. Furthermore, the comparative fitting performance of these three models is comprehensively illustrated in Figure 5.

**Table 3:** Comparison of prediction results of five models.

**Month**	**Incidence**	**SARIMA forecast**		**KNN forecast**		**SARIMA-KNN forecast**		**Prophet forecast**		**SARIMA-Prophet forecast**	
		**Forecast (95% CI)**	**Forecast error (%)**	**Forecast (95% CI)**	**Forecast error (%)**	**Forecast (95% CI)**	**Forecast error (%)**	**Forecast (95% CI)**	**Forecast error (%)**	**Forecast (95% CI)**	**Forecast error (%)**
2024/1/1	1.16	1.40 (−0.33, 3.13)	20.74%	3.31 (0.93, 5.69)	185.39%	1.28 (−0.43, 2.99)	10.69%	1.81 (−0.73, 4.35)	56.10%	1.97 (0.00, 3.94)	69.90%
2024/2/1	1.07	0.86 (−0.87, 2.60)	−19.14%	1.55 (−0.83, 3.93)	45.19%	0.78 (−0.92, 2.49)	−26.42%	−0.42 (−2.96, 2.12)	−139.51%	1.97 (0.01, 3.94)	85.22%
2024/3/1	1.54	1.44 (−0.29, 3.18)	−6.06%	1.37 (−1.01, 3.75)	−10.76%	1.36 (−0.34, 3.07)	−11.11%	0.05 (−2.48, 2.59)	−96.59%	2.87 (0.91, 4.84)	87.15%
2024/4/1	1.41	1.07 (−0.67, 2.80)	−24.20%	1.37 (−1.01, 3.75)	−2.83%	1.00 (−0.70, 2.71)	−28.77%	0.88 (−1.66, 3.42)	−37.65%	1.80 (−0.17, 3.76)	27.42%
2024/5/1	1.79	2.08 (0.35, 3.82)	16.53%	1.55 (−0.83, 3.93)	−13.40%	2.00 (0.30, 3.71)	12.18%	2.26 (−0.28, 4.80)	26.55%	1.56 (−0.41, 3.53)	−12.70%
2024/6/1	1.41	3.22 (1.48, 4.95)	128.05%	1.55 (−0.83, 3.93)	9.70%	3.10 (1.39, 4.81)	119.79%	2.55 (0.01, 5.09)	80.78%	3.14 (1.18, 5.11)	122.94%
2024/7/1	1.88	2.75 (1.01, 4.48)	46.14%	1.55 (−0.83, 3.93)	−17.73%	2.67 (0.96, 4.38)	42.01%	2.13 (−0.40, 4.67)	13.40%	2.82 (0.85, 4.78)	49.80%
2024/8/1	2.76	2.20 (0.47, 3.94)	−20.18%	1.80 (−0.58, 4.18)	−34.67%	2.12 (0.41, 3.83)	−22.99%	0.41 (−2.13, 2.94)	−85.20%	3.28 (1.32, 5.25)	19.10%
2024/9/1	2.07	3.25 (1.52, 4.99)	57.25%	3.07 (0.69, 5.45)	48.42%	3.17 (1.47, 4.88)	53.50%	1.42 (−1.11, 3.96)	−31.25%	4.83 (2.86, 6.80)	133.66%
2024/10/1	0.81	1.83 (0.09, 3.56)	124.53%	2.99 (0.61, 5.37)	267.47%	1.71 (0.00, 3.42)	110.22%	1.07 (−1.46, 3.61)	31.72%	2.83 (0.86, 4.80)	247.76%

SARIMA, seasonal autoregressive integrated moving average; KNN, K-nearest neighbors; Prophet, Facebook Prophet, an additive regression model developed by Meta for time series forecasting; CI, confidence interval.

## 4 Discussion

Our findings demonstrate that the incidence of AHC in Chongqing manifests pronounced seasonal variations, with the peak prevalence consistently observed between June and September annually. This epidemiological phenomenon is strongly associated with the region's distinctive summer climatic conditions. Specifically, the average temperature in Chongqing during this period ranges from 25°C to 30°C, accompanied by relative humidity levels exceeding 70%. Such environmental parameters create optimal conditions for the survival and proliferation of conjunctivitis viruses, consistent with the established biological characteristic that these pathogens thrive in warm and humid environments (8). In addition, under high temperature and humidity conditions during summer, the sebum secreted by the meibomian glands of the eyes increases, creating a more suitable environment for viral propagation. The period from June to September aligns with school summer vacations, during which students engage in various group activities including training programs and summer camps. As a major tourist destination, Chongqing experiences substantial population concentration and high mobility rates. The emergence of even a single case significantly elevates the risk of rapid disease transmission within the community.

The SARIMA-KNN hybrid model developed in this study successfully identified the incidence pattern of AHC in Chongqing. The RMSE of the hybrid model was reduced by 5.9% compared to the single SARIMA model. To further validate the efficacy of hybrid models, we incorporated the Prophet model and its hybrid version with SARIMA as benchmark comparisons. While the Prophet model demonstrates automatic handling of seasonality and holiday effects, its RMSE of 1.39 in this study remains higher than that of the SARIMA-KNN combined model. This finding suggests that although Prophet effectively captures prominent periodic patterns, the KNN's instance-based learning with local adaptability proves more advantageous in processing the complex non-linear residuals in AHC incidence rates. This outcome aligns closely with the core concept of recent monkeypox virus prediction research (33). By constructing structurally appropriate combined models that effectively integrate the strengths of different algorithms, we can overcome the limitations of individual models in characterization capabilities, thereby enabling more comprehensive and precise capture of the complex epidemiological characteristics of infectious diseases. In traditional time series forecasting, the SARIMA model, compared to the ARIMA model, incorporates seasonal effects and has been widely applied in predicting infectious diseases such as influenza and hand-foot-and-mouth disease due to its capability to effectively capture seasonal and periodic patterns (17, 34). However, the SARIMA model demonstrates limited adaptability to sudden events and requires differencing to stabilize non-stationary series, which may result in information loss and reduced prediction accuracy. This limitation has been previously noted in influenza virus prediction studies (35). Conversely, the KNN algorithm has demonstrated remarkable flexibility in non-linear pattern recognition, as evidenced by its applications in air quality prediction and emergency department volume forecasting (36), but it is difficult to handle cyclical patterns when used alone (37). In this investigation, the synergistic integration of both methodologies enabled the SARIMA module to analyze the long-term downward trend and linear patterns of AHC's summer cyclical peak, while the KNN module identified non-linear anomalous fluctuations potentially induced by environmental variations and social behaviors through localized similarity searches, thereby further minimizing prediction errors.

Furthermore, while numerous studies have integrated machine learning with traditional time series models (38, 39), the innovation of our approach resides in the recognition that SARIMA prediction residuals contain spatio-temporal heterogeneity information beyond its linear assumptions. The KNN algorithm performs spatial interpolation on these residuals by incorporating distance weights, thereby achieving geographical refinement of prediction outcomes. In comparison to the widely adopted hybrid models such as SARIMA-LSTM and SARIMA-XGBoost in recent studies (40, 41), the SARIMA-KNN hybrid model proposed in this research demonstrates superior performance in multiple aspects. Not only does it maintain comparable prediction accuracy, but it also exhibits enhanced computational efficiency and improved interpretability. While LSTM models are capable of capturing complex sequential dependencies, they necessitate substantial training data and intricate hyperparameter optimization. Similarly, despite its robust predictive capabilities, XGBoost presents interpretability challenges regarding epidemiological mechanisms due to its inherent “black box” characteristics. Conversely, KNN offers distinct advantages, including an intuitive algorithmic principle, minimal parameter requirements, and superior adaptability to medium and small-scale datasets. These attributes render it particularly valuable for infectious diseases with limited data availability, such as AHC, or in resource-constrained regions. Furthermore, given the abrupt onset and rapid transmission patterns characteristic of AHC outbreaks, which demand swift public health responses, the SARIMA-KNN hybrid model's prediction outputs can be seamlessly integrated with existing infectious disease surveillance network reporting systems. This integration facilitates the development of a dynamic early warning system (42). Through comparative analysis of short-term predicted values against historical baseline levels and the establishment of multi-tiered risk-level warning thresholds, the system can automatically generate alerts to disease control authorities when predicted values surpass predefined thresholds. This automated mechanism enables prompt enhancement of pathogen monitoring and preparation of epidemic prevention materials, thereby optimizing prevention and control efficiency.

Although the SARIMA-KNN hybrid model in this study was developed for predicting the incidence of acute hemorrhagic conjunctivitis, its methodology can be extended to disease prediction and emergency response scenarios. Similar to the ANN-CVD study (43) that utilized artificial neural networks to predict cardiovascular disease mortality in Pakistan, this model, by combining SARIMA and KNN, is also suitable for health data that requires consideration of both long-term trends and short-term fluctuations. Moreover, the response to public health emergencies such as COVID-19 needs to take into account policy dynamics and time sensitivity (44). The hybrid framework of SARIMA-KNN model can be enhanced by integrating external covariates and optimizing the sliding window, providing a time-sensitive predictive tool for emergency decision-making. This direction is highly consistent with the goal of building a multi-disciplinary intelligent early warning system. Future research should introduce feature importance assessment methods (45) to clarify the contribution of external variables such as meteorology and population mobility to the prediction results, and focus on constructing a multi-disciplinary integrated intelligent prediction system. It is recommended to develop adaptive hybrid models (such as SARIMA-Transformer-XGBoost) to enhance the response capability to public health emergencies. At the same time, a spatio-temporal dynamic early warning platform can be established to achieve county-level risk classification and optimal resource allocation (46). Relevant institutions should enhance the medical data sharing mechanism and privacy protection standards, promote the inclusion of prediction models in local infectious disease prevention and control guidelines. Ultimately, a closed-loop management system encompassing “data-driven—model prediction—decision support” should be established to provide intelligent solutions for the scientific prevention and control of climate-sensitive infectious diseases, such as acute hemorrhagic conjunctivitis.

## 5 Limitation

Although the SARIMA-KNN hybrid model proposed in this study demonstrated high accuracy in predicting AHC, several limitations still exist. Firstly, the relatively short time span of the data used in this study restricted the model's ability to capture longer-term epidemic trends or multi-year cyclical patterns. Secondly, the model's generalization ability may be limited by regional characteristics, and its predictive performance may vary in other provinces, cities, or regions. Additionally, the reliability of long-term predictions is constrained by the assumption of stability in environmental and social factors. If extreme climates or policy adjustments occur in the future, the prediction results may deviate. These limitations suggest that it is necessary to continuously accumulate longer time series of monitoring data and integrate multi-source data to optimize the model structure, thereby enhancing the applicability of the prediction. Future research should also focus on collecting incidence data of AHC in other geographical areas and conducting external validation of this model to comprehensively evaluate its robustness and applicability across regions.

## 6 Conclusion

This study pioneers the introduction and validation of a SARIMA-KNN hybrid model, employing a residual correction strategy for the prediction of AHC in Chongqing. Comparative analyses with SARIMA, KNN, Prophet models, and the SARIMA-Prophet hybrid model demonstrate the superior performance of the SARIMA-KNN hybrid model across multiple error metrics (MSE, MAE, RMSE, and MAPE), achieving significant reductions in prediction error. The research not only identifies the seasonal characteristic that the period from June to September constitutes the peak incidence of AHC but also offers a practical tool for public health decision-making, thereby enhancing the accuracy and real-time performance of existing infectious disease early warning and monitoring platforms. Future research directions may focus on enhancing the model performance through integrating multi-source data and optimizing the deep learning architecture. Furthermore, it is recommended to connect the model output with the regional prevention and control resource scheduling system to achieve precise early intervention for infectious diseases.

## Data Availability

The original contributions presented in the study are included in the article/supplementary material, further inquiries can be directed to the corresponding author.
